# Color Recurrence Plots for Bearing Fault Diagnosis

**DOI:** 10.3390/s22228870

**Published:** 2022-11-16

**Authors:** Vilma Petrauskiene, Mayur Pal, Maosen Cao, Jie Wang, Minvydas Ragulskis

**Affiliations:** 1 Department of Mathematical Modelling, Kaunas University of Technology, Studentu 50-146, LT 51368 Kaunas, Lithuania; 2 Department of Engineering Mechanics, Hohai University, Hohai 210098, China; 3 College of Civil and Architecture Engineering, Chuzhou University, Chuzhou 239000, China; 4 Intelligent Transportation and Intelligent Construction Engineering Research Center, Jiangsu Dongjiao Intelligent Control Technology Group Co., Nanjing 211161, China

**Keywords:** recurrence plot, nonuniform embedding, transfer learning, feature extraction, bearing fault diagnosis

## Abstract

This paper presents bearing fault diagnosis using the image classification of different fault patterns. Feature extraction for image classification is carried out using a novel approach of Color recurrence plots, which is presented for the first time. Color recurrence plots are created using non-linear embedding of the vibration signals into delay coordinate space with variable time lags. Deep learning-based image classification is then performed by building the database of the extracted features of the bearing vibration signals in the form of Color recurrence plots. A Series of computational experiments are performed to compare the accuracy of bearing fault classification using Color recurrence plots. The standard bearing vibration dataset of Case Western Reserve University is used for those purposes. The paper demonstrates the efficacy and the accuracy of a new and unique approach of scalar time series extraction into two-dimensional Color recurrence plots for bearing fault diagnosis.

## 1. Introduction

Any rotating equipment, which is part of heavy machinery, or comprising of mechanical motors employs rolling or other types of ball bearings as one of the key elements for the purpose of uni- or multi-dimensional rotations. Repeated use of the bearing and excessive vibrations, combined with limited lubrication of machine parts could sometimes lead to the development of mechanical faults in the bearing. These mechanical faults if gone undetected for some time could result in machine failure, consequently resulting in downtime and sometimes could also lead to injuries. Timely interventions or preventive maintenance is key to keeping high up-time of the rotating equipment. One way to ensure that preventive maintenance could be performed is if an early bearing fault detection could be available. Early fault detection is not easy as it is sometimes hard to nearly impossible to implement non-intrusive inspections of heavy machinery due to inaccessibility. Intrusive inspections are not always welcome as they could result in heavy downtime and impact production. Non-intrusive inspection is the preferred route to limit downtime. One way to perform the non-intrusive inspection is through the use of vibration data from the machine and analyzing it with help of machine learning algorithms.

Machine learning algorithms have been successively used for early fault detection in rotational bearings. Tools such as support vector machines and artificial neural networks have been used in feature extraction for bearing fault diagnosis [1]. An image classification approach which results in a confusion matrix as an evaluation parameter is employed. A review of different artificial algorithms used in fault diagnosis can be found in [2,3,4]. A brief overview of the main artificial intelligent architecture used for bearing fault diagnosis is discussed in [5].

Advanced ANN architectures are often utilized for bearing fault identification and classification [6,7,8]. Bearing fault analysis has been carried out using permutation entropy approaches and has been discussed in great detail in the paper [9,10]. Recurrence analysis has also been employed in bearing fault diagnosis using uniform time delays, see paper [11,12]. Some other authors have also tried to apply Recurrence Quantification analysis for Bearing Fault detection [13]. Deep learning-based bearing fault analysis has been extensively looked at [1]. Fault diagnosis of bearings using recurrences based on uniform time delays and artificial intelligence techniques, such as rotation forest, artificial neural network, and support vector machine, has also been looked at recently by [14]. Supervised and unsupervised methods for fault diagnosis using feature representation have also been investigated [15,16]. The capability of recurrence plots to extract visual features from a scalar time series has been extensively exploited in deep learning-based algorithms for intelligent fault diagnosis. For example, recurrence plots with optimal time delay have been used in [11] for quantitative analysis of fault diagnostic. The recurrence plot-based damage method was also introduced in [12]. Although deep learning has been used in bearing fault diagnosis, a comprehensive review of the literature published in the field of bearing fault diagnosis, using deep learning, presented in [17] suggest that the transfer learning approach should be explored in bearing fault diagnosis.

One area where there is still a large gap is the use of non-uniform time delay space combined with colored recurrence analysis and deep learning. The literature in this area is largely missing to the best of the authors’ knowledge. Our paper addresses this gap. In this paper, we plan to study the ability of deep-learning CNN models to automatically learn the useful texture features in order to classify bearing faults. One-dimensional raw current signals are converted to 2D images, and the CNN model is used to successfully capture the temporal and spatial dependencies in the colored images, which are generated using recurrence analysis.

The main objective of this paper is to propose Color recurrence plots as a new feature extraction technique for bearing fault diagnosis. Color recurrence plots are capable to extract visual features from a vibration signal represented in the form of a scalar time series. The paper demonstrates that the performance of fault classification is considerably improved by the combined implementation of feature extraction using Color recurrence plots, and the CNN classifier. The paper also highlights the role of non-uniform embedding in the generation of the database of images used for training the machine learning model. Since the main aim of this paper is to introduce novel feature extraction techniques; therefore, the transfer learning approach is used for training two different trained networks, namely Alexnet and SqueezeNet. The networks are chosen on the basis of their computational cost and accuracy. A detailed comparison is presented in the paper using these two networks for bearing fault diagnosis.

The paper is presented as follows: The Introduction is presented in Section 1. Section 2 describes the non-uniform embedding approach and principles behind recurrence plot Creation. Definitions of the proposed Color recurrence plot analysis approach are presented in Section 3. Details of the data set used in the paper are described in Section 4. Section 5 describes the networks used for transfer learning and the input data requirements for training and testing of these networks for the Color recurrence plot-based classification task is presented. Results from the application of transfer learning for Color recurrence analysis are presented in Section 6. Conclusions and discussions follow in Section 7.

## 2. Preliminaries

In this section, the basic building blocks for the fault diagnostic algorithm presented in this paper are discussed. First, the principles of non-uniform time-delay embedding are presented followed by details of recurrence plot concepts for feature extraction are introduced.

### 2.1. Non-Uniform Embedding

Let us consider a scalar time series on a regular grid:

(1) $$\begin{array}{c} \left\{x_{k}\right\},k=1,2,\ldots ,N;x_{k}\in R \end{array}$$

where *N* is the length of the observation window. Uniform embedding maps the time series into a trajectory matrix:

(2) $$\begin{array}{c} \left[\begin{array}{ccccc} x_{k} & x_{k+\tau } & x_{k+2\tau } & \cdots & x_{k+\left(d-1\right)\tau } \end{array}\right];\\ k=1,2,\ldots ,\left(N-\left(d-1\right)\tau \right), \end{array}$$

where *d* is the embedding dimension and τ∈N is the time lag. One of the classical methods used for the determination of the optimal embedding dimension is the false nearest neighbor (FNN) method [18]. The identification of the optimal time lag is performed by using algorithms based on the auto-correlation function [19], the mutual information [20], or the geometric approach where the optimality of the time delay is based on the maximal spreading of the embedded attractor in the delay-coordinate space [21]. In general, the optimal time lag should make column vectors of the trajectory matrix to be independent as far as possible, yet not too far to preserve the information about the dynamic properties of the embedded time series.

Non-uniform attractor embedding also maps the original time series into a trajectory matrix:

(3) $$\begin{array}{c} \left[\begin{array}{ccccc} x_{k} & x_{k+\tau_{1}} & x_{k+\tau_{1}+\tau_{2}} & \cdots & x_{k+\tau_{1}+{\cdots +\tau }_{d-1}} \end{array}\right];\\ k=1,2,\ldots ,\left(N-\left(\tau_{1}+{\cdots +\tau }_{d-1}\right)\right), \end{array}$$

when time lags $\tau_{1}$, $\tau_{2}$, …, $\tau_{d-1}\in N$ are not necessarily equal. It has been demonstrated that non-uniform embedding has many advantages over uniform embedding. For example, it is shown in [19] that non-uniform embedding is preferable for the reconstruction of attractors in a multi-dimensional delay coordinate space when a time series involves several incommensurate frequencies. It is demonstrated that non-uniform embedding does outperform uniform embedding in time series forecasting applications [22,23]. Non-uniform embedding is efficiently exploited for the detection of the causal coupling and the transfer entropy in multivariate time series [24,25]. 

Many different techniques exist for the determination of the set of optimal time lags $\left\{\tau_{1},\;\tau_{2},\;\ldots ,\;\tau_{d-1}\right\}$. Evolutionary algorithms for the selection of time lags based on the near-optimal spreading of the reconstructed attractor in all possible projections of the delay coordinate space are employed in [26]. A greedy strategy for constructing the embedded vector based on direct-coupling information measure is presented in [27]. The conditional entropy criteria and greedy forward selection are exploited for nonuniform embedding in [28]. The feature selection technique, in which the objective function of nonuniform embedding is based on the relevance analysis, is presented in [29]. A pure geometric approach for the determination of optimal time lags of the non-uniform embedding is presented in [30]. This approach is based on the maximization of the following target function *T*:

(4) $$\begin{array}{c} \underset{1\leq \tau_{1},\ldots ,\tau_{d-1}\leq m}{max}T\left(\tau_{1},\tau_{2},\ldots ,\tau_{d-1}\right)=\frac{1}{\left(N-\delta \right)\sqrt{d}}\\ \times \sum_{k=1}^{N-\delta }\sqrt{x_{k}^{2}+x_{k+\tau_{1}}^{2}+x_{k+\tau_{1}+\tau_{2}}^{2}+\cdots +x_{k+\delta }^{2}}\; \end{array}$$

where δ is the length of the embedding window: $\delta =\sum_{k=1}^{d-1}\tau_{k}$, and *m* is the upper limit for the time lags. This is a simple, straightforward, and efficient method for the determination of the optimal set of time lags for a scalar time series in a finite observation window [30]. We will use this method for the determination of non-uniform time lags in this paper.

### 2.2. The Standard Recurrence Plot

Dynamical systems may exhibit recurrence relationships in some time-space coordinate systems. A trajectory generated by a dynamical system does repeat itself at some time interval τ through a phase space [31]. The recurrence plot is a medium to visualize such a relationship in 2D.

The recurrence plot shows the distance of every data point $x_{t_{i}}$, at time $t_{i}$, to all other points in the phase space. Explicitly, this distance is given by $D\left(i,j\right)=∥\left(X_{i}-X_{j}\right)∥)$. The value of the recurrence plot at coordinates (i,j) is set to 1 if $∥\left(X_{i}-X_{j}\right)∥)$ is less than a given threshold parameter ε, and is set to 0 otherwise. The recurrence plot obtained this way is a dichotomous plot of black and white pixels. The selection of the threshold parameter ε is crucial for building a meaningful and representative recurrence relationship. If ε is too small then there is almost no recurrence relationship and we would not be able to learn anything about the recurrence relationship of the dynamical system. Otherwise, if ε is too large then as well it would result in a recurrence relationship which may mask the true behavior of the dynamical system. Usually, ε is chosen in such a way that the proportions of white and black pixels in the recurrence plot are equal [30].

Mathematically the recurrence relationship is expressed as the collection of pairs of indexes at which the trajectory is at the same place:

(5) $$\begin{array}{c} R\left(i,j\right)=\left\{\begin{array}{c} 1\;if\;\;|x_{i}-x_{j}|⩽\varepsilon ;\\ 0\;otherwise,\;\;\;\;\;\;\;\;\;\;\; \end{array}\right. \end{array}$$

where indexes i,j sweep over the time series producing a square symmetric digital image: 1≤i,j≤N. Recurrence plots are beneficial to visualize the recurrent dynamics of a time series and can be used for the distinction of periodic, quasi-periodic, chaotic, and random time series [32].

Although the concept of the recurrence plot was introduced by Eckman in 1987 [31], over time it has become a powerful method for graphical representation and analysis of nonlinear time series.

**Figure 1:**
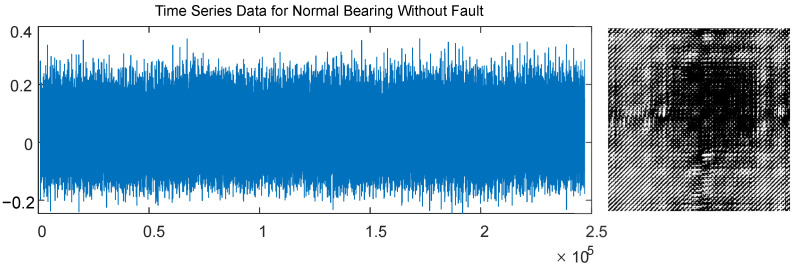
The standard recurrence plot generated by a normal bearing vibration signal without a fault (the dataset of Case Western Reserve University School of Engineering [33]). The threshold parameter ε is set to 0.253 which results in an equal proportion of black and white pixels.

A standard recurrence plot generated by a normal bearing vibration signal without a fault is shown in Figure 1 (bearing vibration signal is retrieved from the standard dataset of the Case Western Reserve University Bearing Data Center database [33]). The threshold parameter ε used for the construction of the recurrence plot in Figure 1 is set to 0.253 (that results in an equal proportion of black and white pixels).

**Figure 2:**
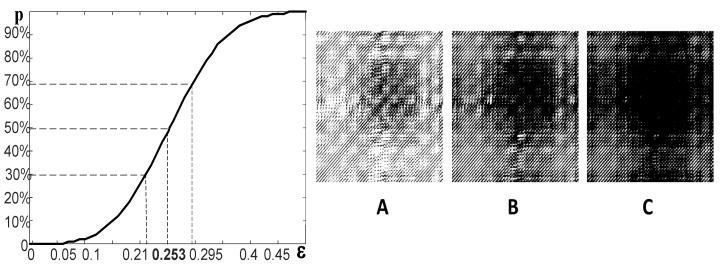
The standard recurrence plot of a normal bearing vibration signal without a fault. The line graph shows the relationship between the percentage of black pixels in the recurrence plot from the threshold parameter ε. The ratio between black and white pixels is equal at ε=0.253. Part (**A**) shows the standard recurrence plot at ε=0.21; part (**B**) at ε=0.144; part (**C**) at ε=0.295.

The selection of this particular value of ε is illustrated in Figure 2. The ratio between black and white pixels is 30% at ε=0.21 (Figure 2A); the ratio between black and white pixels is 70% at ε=0.295 (Figure 2C). The graphical relationship between the ratio of pixels and ε is a monotonous sigmoidal-type function (Figure 2). A proper selection (optimization) of ε helps to generate a representative standard recurrence plot.

### 2.3. Recurrence Plots for Bearing Fault Diagnosis

A recurrence plot can be interpreted as a feature extraction algorithm from a time series. A scalar time series is mapped onto a 2D digital image through the recurrence relationship. That enables the use of machine learning algorithms for the classification of features (digital images) extracted from the original time series.

A typical engineering application of recurrence plots is the bearing fault diagnosis. A standard dataset used for such purposes is the Case Western Reserve University Bearing Data Center database [33]. The database has become a standard test set for intelligent defect detection algorithms in rolling bearings systems. The data come from the experimental test rig comprised of a 2-hp motor, a torque sensor/encoder, a power meter, accelerometers, and electronic control unit. The faults are created by electrical discharge machining. There are four different health states of rolling bearings—the inner race fault, the outer race fault, the ball fault, and no fault. Vibration data are classified into fault data when the defect is on the drive end (sampled at 12k), fault data when the defect is on the drive end (sampled at 48k), fault data when the defect is on the fan end, and normal baseline data [33]. Several authors have already successfully used standard recurrence plots for the classification of bearing faults [11,12].

## 3. The Color Recurrence Plot

The main objective of this paper is to introduce the concept of the Color recurrence plot. Non-uniform embedding plays a major role in the algorithm generating the Color recurrence plot.

### 3.1. The Proposed Recurrence Plot Based on a Fixed Time Lag

**Figure 3:**
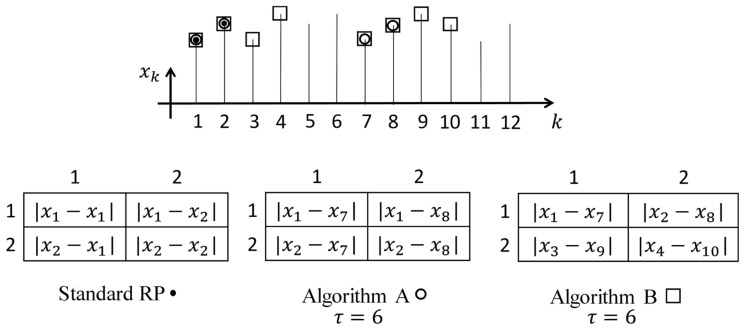
Figure showing 3 different recurrence plot generation algorithms. Standard recurrence plot, Algorithm A corresponds to a recurrence relationship involving non-uniform time delay with time delay τ +/− 1, Algorithm B corresponds to the recurrence relationship involving purely non-uniform time delays τ.

Without loss of generality, let us consider a 2×2 standard recurrence plot (Figure 3). The elements of the standard recurrence plot in Figure 3 are marked as moduli of differences between the data points according to the indexes of the row and the column (the comparison to the threshold parameter ε is omitted for brevity). Only the first two data points of the time series $x_{1}$ and $x_{2}$ are used in the formation of the standard recurrence plot. These two first data points are marked by black dots in the schematic representation of the time series (Figure 3).

Let us consider a two-dimensional embedding of the time series into a planar phase plane. The algorithm for the construction of the standard recurrence plot could be extended in order to represent the process of two-dimensional embedding in several different ways. If the time lag used for time series embedding is τ, then the index of the second element in the modified recurrence plot can be set increased by τ. Such an approach is illustrated by Algorithm A in Figure 3. Note that Algorithm A does not produce a symmetric matrix. The number of data points used for the formation of the modified 2×2 recurrence plot according to Algorithm A is equal to four (the data points are marked as empty circles in Figure 3).

However, the difference between indexes of data points in the modified recurrence plot generated by Algorithm A is not always equal to τ. The difference between indexes is equal to 5, 6, and 7 in the 2×2 modified recurrence plot at τ=6 (Figure 3). Note that the variation interval of those differences around τ will be much longer if the size of the modified recurrence plot is larger. In other words, the information about the pre-selected time lag τ will be kept only on the main diagonal of the modified recurrence plot.

As mentioned previously, the concept of the Color recurrence plot will be closely related to the non-uniform multi-dimensional embedding. The set of optimal time lags does comprise d−1 time lags (when the dimension of the delay-coordinate space is *d*). It is important to keep particular values of time lags fixed during the embedding process. Therefore, we do introduce Algorithm B (Figure 3) which will be used as the main building block in the process of the construction of the Color recurrence plot.

Algorithm B is directly related to the geometric representation of the embedding process—the pre-selected time lag τ is kept unchanged during the whole embedding process. The number of elements in the 2×2 proposed recurrence plot is 4 (Figure 3). The index of the first data point in the difference runs consecutively through the time series starting from the first data point (Algorithm B). The index of the second data point in the difference is lagged strictly by τ (Figure 3). Eight data points are used for the formation of the proposed 2×2 recurrence plot according to Algorithm B; those data points are marked by empty rectangles in (Figure 3).

Note that the proposed recurrence plot produced by Algorithm B also produces a dichotomous digital image (the pixels are either black or white). Moreover, the proposed recurrence plot does not need to be a square matrix; there are no restrictions for the dimension of the generated plot (except the length of the time series).

**Figure 4:**
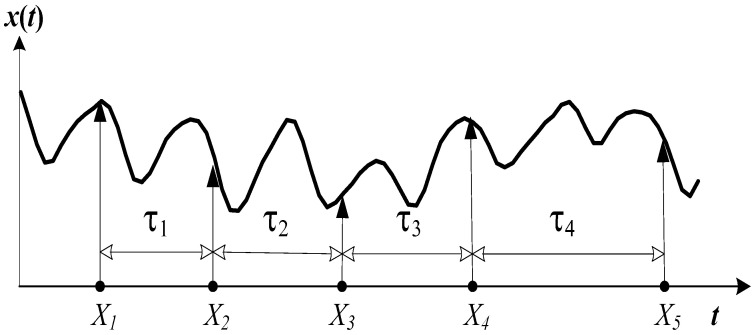
A schematic diagram illustrating non-uniform embedding of a scalar time series into a 5-dimensional delay-coordinate space. $X_{1},\ldots ,X_{5}$ denote the axes of the reconstructed phase space; $\tau_{1},\ldots ,\tau_{4}$ denote time delays.

Let us denote the axes of the delay-coordinate space as $X_{1},\ldots ,X_{d}$ (Figure 4). Then, the total length of the embedding window is $\tau_{1}+\tau_{2}+\cdots +\tau_{d-1}$; the number of rows of the trajectory matrix (3) is:

(6) $$\begin{array}{c} L=n\cdot m\leq N-\left(\tau_{1}+\cdots +\tau_{d-1}\right), \end{array}$$

Let us define the size of the binary image representing the recurrence plot based on a fixed time delay as n×m. Let us choose a planar projection generated by coordinate axes $X_{k}$ and $X_{l}$; 1≤k,l≤d−1.

Note that the time lag $\delta_{kl}$ between axes $X_{k}$ and $X_{l}$ is (Figure 4):

(7) $$\begin{array}{c} \delta_{kl}=\tau_{k}+\cdots +\tau_{l-1};1\leq k<l\leq d. \end{array}$$

Therefore, the recurrence plot in the planar projection generated by $X_{k}$ and $X_{l}$ reads:

(8) $$\begin{array}{c} P_{kl}^{\left(n\times m\right)}\left(i,j\right)=\left\{\begin{array}{c} 1\;if\;\;|x_{p_{i}}-x_{p_{j}}|⩽\varepsilon ;\\ 0\;otherwise,\;\;\;\;\;\;\;\;\;\;\; \end{array}\right. \end{array}$$

where $p_{i}=\left(i-1\right)m+j$; $p_{j}=p_{i}+\delta_{kl}$; 1≤k<l≤d; 1≤i≤n; 1≤j≤m. Note that the time lag between indexes $p_{i}$ and $p_{j}$ is always kept constant.

### 3.2. The Color Recurrence Plot Based on Non-Uniform Embedding

Let us consider that the dimension of the delay-coordinate space is d>2. In other words, a scalar time series is embedded into a *d*-dimensional space. The properties of the embedded attractor can be evaluated by assigning a measure function for the attractor’s planar projection. Then, the values of the measure function produced by all possible planar projections in the *d*-dimensional phase space can be averaged. The resulting value can be used as a numerical estimate of the properties of the embedded attractor [23]. By the way, the measure function used in [23] is the area occupied by the attractor in a planar projection.

The proposed Color recurrence plot is constructed using the same principle of arithmetic averaging throughout all possible planar projections of the *d*-dimensional phase space. However, the measure function used to construct the proposed Color recurrence plot does not map the projection of the attractor into a single scalar value (as used in [23]). The proposed measure function μ maps the projection of the embedded attractor (the *k*-th and the *l*-th columns of the trajectory matrix) into $P_{kl}^{\left(n\times m\right)}$:

(9) $$\begin{array}{c} \mu \left(X_{k},X_{l}\right):R^{L\times 2}\to P_{kl}^{\left(n\times m\right)}. \end{array}$$

The number of different planar projections in the d-dimensional phase space is $\frac{d(d-1)}{2}$. The arithmetic averaging through all possible planar projections yields the Color recurrence plot:

(10) $$\begin{array}{c} C^{\left(n\times m\right)}=\frac{2}{d(d-1)}\sum_{1\leq k,l\leq d;k\neq l}^{}P_{kl}^{\left(n\times m\right)}. \end{array}$$

The maximal number of different colors in the Color recurrence plot is $\frac{d(d-1)}{2}+1$. This number exceeds 256 only at d=23. Therefore, the different colors of $C^{\left(n\times m\right)}$ can be linearly distributed throughout the standard grayscale interval [0,255].

### 3.3. Color Recurrence Plots Produced by Bearing Vibration Signals

Let us consider a normal ball-bearing vibration signal without a fault (the same signal used in Figure 1). The first step in the process of building the Color recurrence plot is the determination of the optimal embedding dimension *d*. The classical FNN algorithm [34] yields d=5 for this vibration signal.

**Figure 5:**
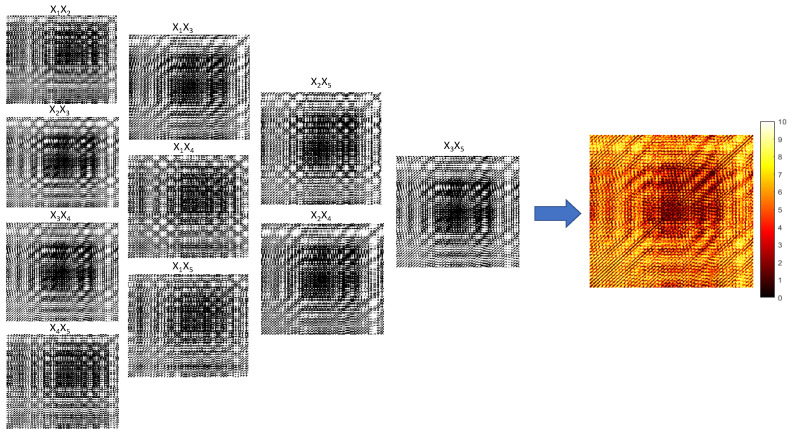
A schematic diagram illustrating the formation of the Color recurrence plot for a normal bearing test data without a fault. The embedding dimension d=5 yields ten different dichotomous recurrence plots based on a fixed time lag. The first column represents recurrence plots with a single time delay ($\tau_{1}$, $\tau_{2}$, $\tau_{3}$, and $\tau_{4}$). The second column represents recurrence plots with double time delays ($\tau_{1}+\tau_{2}$, $\tau_{2}+\tau_{3}$, and $\tau_{3}+\tau_{4}$). The third column represents triple time delays ($\tau_{1}+\tau_{2}+\tau_{3}$, and $\tau_{2}+\tau_{3}+\tau_{4}$). The fourth column represents a recurrence plot with the maximal time delay ($\tau_{1}+\tau_{2}+\tau_{3}+\tau_{4}$). The Color recurrence plot is produced by the arithmetic averaging of dichotomous plots; the number of different colors is 11. The Color recurrence plot is enlarged for clarity.

The maximization of the target function (4) yields the following set of optimal time delays: $\tau_{1}=5$; $\tau_{2}=39$; $\tau_{3}=5$, and $\tau_{4}=15$. Ten different recurrence plots based on the fixed time delay (corresponding to ten different projections of the embedded attractor) are depicted in Figure 5. The threshold parameter ε is set to 0.253 for all ten recurrence plots. The arithmetic average of ten dichotomous digital images yields the Color recurrence plot (Figure 5).

Ten recurrence plots based on a fixed time delay (corresponding to ten different projections of the embedded attractor) are depicted in Figure 5. All ten dichotomous recurrence plots are grouped into four columns. The first column represents recurrence plots with a single time delay ($\tau_{1}$, $\tau_{2}$, $\tau_{3}$, and $\tau_{4}$). The second column represents recurrence plots with double time delays ($\tau_{1}+\tau_{2}$, $\tau_{2}+\tau_{3}$, and $\tau_{3}+\tau_{4}$). The third column represents triple time delays ($\tau_{1}+\tau_{2}+\tau_{3}$, and $\tau_{2}+\tau_{3}+\tau_{4}$). Finally, the fourth column represents a single recurrence plot with the maximal time delay in the five-dimensional delay coordinate space ($\tau_{1}+\tau_{2}+\tau_{3}+\tau_{4}$).

The Color recurrence plot is generated by computing the arithmetic average of all ten dichotomous recurrence plots based on a fixed time lag; the number of different colors is $\frac{5\cdot 4}{2}+1=\;11$ (marked from 0 to 10 in Figure 5). Note that the size of dichotomous recurrence plots and the Color recurrence plot in Figure 5 is the same (the Color recurrence plot is enlarged for clarity).

**Figure 6:**
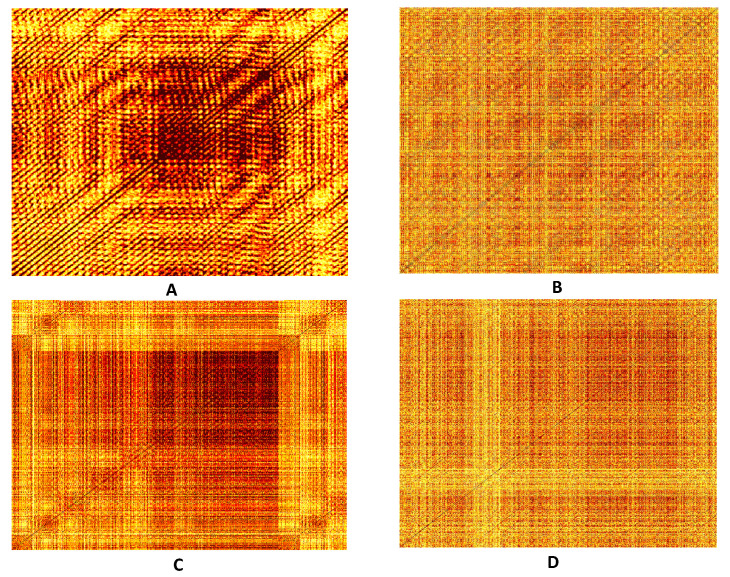
Color recurrence plots generated by vibration signals of a ball bearing. Part (**A**) shows the Color recurrence plot produced by a normal bearing without a fault. Parts (**B**–**D**) show recurrent plots generated by the bearing with the ball fault, the inner race fault, and the outer race fault accordingly.

Color recurrence plots generated by vibration signals recorded on the normal bearing, the bearing with a ball fault, the bearing with an inner race fault, and the bearing with an outer race fault are depicted in Figure 6. A naked human eye can see clear visual differences between the four digital images. That is a good indication that the proposed feature extraction algorithm is able to differentiate between different working conditions of the ball bearing.

## 4. The Description of the Data Set

The data set used in this work comes from the Case Western Reserve University School of Engineering [33]. The data corresponds to ball bearing test data for normal and faulty bearings. Data comes from laboratory-based experiments, which were conducted using a 2Hp reliance Electric motor, and acceleration data was measured at locations near to and remote from the motor bearings.

The fault-bearing data set has faults ranging from 0.007 inches to 0.040 inches (the faults are created by an electrical discharge machine). The data corresponding to the 0.007-inch fault is used in our study. We use the least significant faults only to demonstrate the sensitivity of the proposed fault diagnosis approach. There are four different health states of the bearings depending on their position relative to the near or far end of the motor. These states are namely, the normal state with no fault, the inner race fault, the outer race fault, and the ball fault. The original data set has data available for varying speeds of the motor ranging from 0 to 3 HP. In our study we have focused on a single motor speed of 0 HP. Again, we are choosing the vibration signals with the least expressed fault sensitivity.

Four sets of Color recurrence plots are generated for vibration signals corresponding to the normal state, the ball fault, the inner race fault, and the outer race fault. Non-overlapping windows are used for the time delays to create averaged recurrence plot for each data set resulting in a total of 221 images each for normal, ball fault, inner race, and outer race fault data sets.

## 5. Transfer Learning for Bearing Fault Diagnosis

The transfer learning approach is used for training, testing, and validation of the machine learning approach used in this paper. Such an approach is used due to several reasons. First, the transfer learning approach allows for the testing of several well-established deep learning networks. Additionally, the transfer learning approach saves time as it eliminates the need for the development of a network from scratch, which is not an easy task and needs network designing expertise. Finally, the transfer learning approach is easy to implement and allows for faster simulation and better sensitivity which could be achieved in a relatively short amount of time in a real-world application scenario.

### 5.1. Transfer Learning Definition

The basic idea of the transfer learning approach is to use a well-trained network and utilize the knowledge it has acquired for another task, which is similar in nature by exposing or training it on some additional set of parameters [35]. A real-world analogy would be to take a person who has programming skills to code in languages such as C_++_ or Java and expose him to programming in python. The person would be able to use the skills acquired in learning C_++_ or Java and would be able to learn python much faster compared to a person who has no previous programming knowledge.

**Figure 7:**
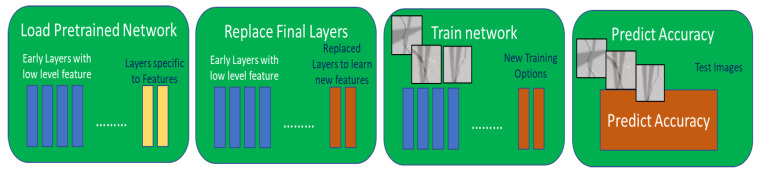
Workflow showing retraining of a pretrained network.

In this paper, since the focus is on image classification, several pretrained image classification networks exist, SqueezeNet [36] and Alexnet [37]. Both networks have been trained to classify over 1000 images. Therefore, in principle, it is possible to take one of these networks and re-train them for a new classification task. This could be achieved by re-training the network on an additional set of images by changing some network parameters to classify a new set of images. Fine-tuning the network parameters of a pretrained network is much faster and easier compared to creating a network from scratch. This is exactly the approach that has been used in this paper. A sample workflow of the approach is shown in Figure 7.

### 5.2. Pretrained Network Selection

**Figure 8:**
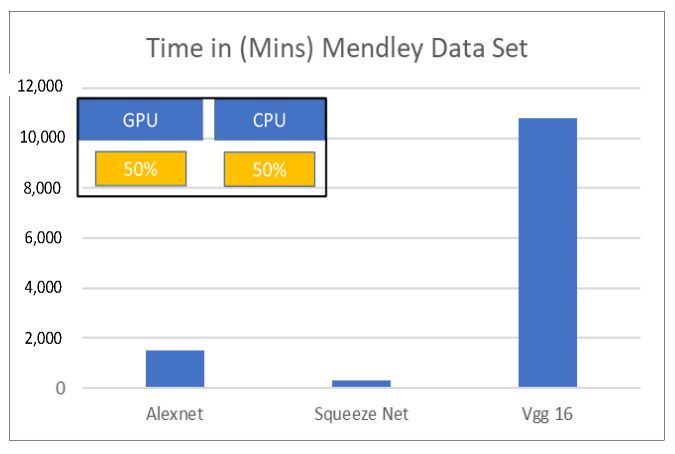
Figure showing a comparison of speed for different classification networks tested on images from the Mendeley DataSet, for details please see [40,41].

The first task in transfer learning is to select the pretrained network to be used for the transfer learning approach. For this purpose, we draw upon a comparison of prediction time vs. prediction accuracy, see Figure 8 taken from [38]. The plots show some pretrained networks, which are very fast but have poor prediction accuracies, such as Squeezenet and some pretrained networks, which are very accurate but take a lot of computational time such as Inception-ResNet-V2 [39].

In the work presented in this paper, we wanted to perform many sensitivities; hence, we required pretrained networks that are fast for initial testing. Since the main aim of this paper is feature extraction and not network engineering, we made a choice of using SqueezeNet and AlexNet due to their fast computational speed.

### 5.3. Transfer Learning for AlexNet and SqueezeNet

Once the pretrained networks are selected transfer learning approach is used to re-train the pretrained networks. In this section, some details of the two networks are provided along with the details of the procedure used for the retraining of the AlexNet and SqueezeNet networks is also provided.

#### 5.3.1. Transfer Learning for AlexNet

AlexNet is an image classification network comprising 25 layers and has been trained to classify up to 1000 images. Input image size for the network is 227 × 227 pixels resolution. For retraining of the AlexNet, the last three layers that are configured for 1000 classes must be fine-tuned for the new classification problem. These last three layers are extracted and replaced by a fully connected layer, a SoftMax layer and a classification layer. Some learning parameters are then adjusted to, e.g., Weightlearnfactor and Biaslearnfactor. The initial learning rate parameter is also adjusted to slow down the learning rate of the new network to increase its prediction capabilities. Now, the new network is retrained with a new data set of images.

#### 5.3.2. Transfer Learning for SqueezeNet

SqueezeNet is an image classification convolutional neural network, which has 18 deep learning layers and a total of 68 neural network layers. The SqueezeNet is also trained to classify up to 1000 classes of images. Input image size for the network is 227 × 227 pixels resolution. The network is trained on a wide range of images and has a rich feature representation. The convolutional layer of the network extract features that the last learnable layer and the final classification layer use to classify the input image. The two layers ‘conv10’ and ‘classificationLayer_preidction’ contain information on how to combine the features that the network uses for class probabilities. For retraining of the SqueezeNet the last two layers are replaced with a new set of layers to adapt to the new data set. In SqueezeNet, the last learnable layer is the final convolutional layer instead, which needs to be replaced by a new convolutional layer with a number of filters equal to 2 for the classification of the new data set with crack and without crack. Some learning parameters are then adjusted to, e.g., Weightlearnfactor and Biaslearnfactor. The initial learning rate parameter is also adjusted to slow down the learning rate of the new network to increase its prediction capabilities. Now, the new network is retrained with a new data set of images.

#### 5.3.3. Network Parameter Optimization

**Figure 9:**
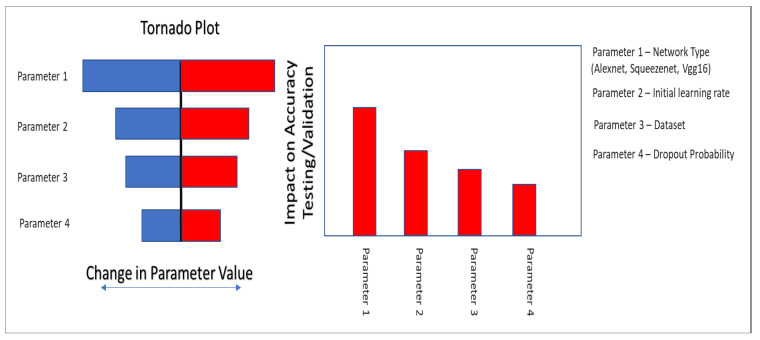
Figure showing a conceptual workflow for network parameter optimization.

In this paper, we have used an experimental design approach to test the impact of network parameters on output accuracy, to help identify, and then change the most impacting network parameters. A conceptual approach for the experimental design workflow is shown in Figure 9. The workflow used is rather simple and straightforward to implement. The steps of the workflow are as follows:

- Identify the most impacting network parameter through a sensitivity analysis.
- For sensitivity analysis, a parameter range is decided and then prediction is performed using the set of network parameter impact on prediction accuracy is checked.
- Several combinations of different network parameters are tested.
- Finally, the network parameters giving the best prediction accuracy are selected

**Figure 10:**
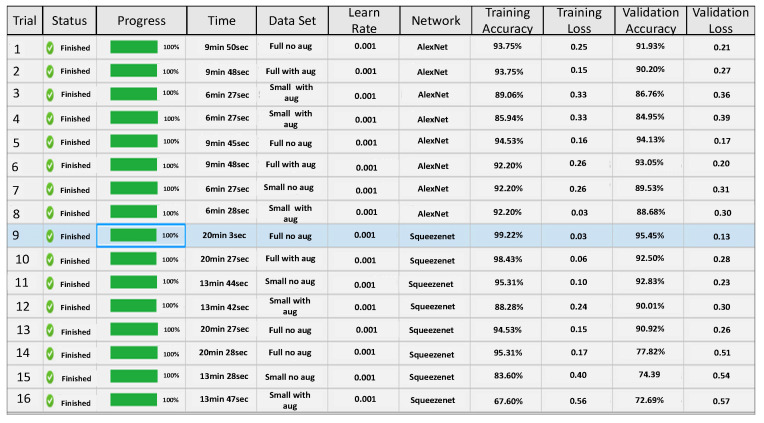
Figure showing application of network optimization workflow on AlexNet and SqueezeNet using the database of images and trial runs for initial learning rateparameter optimization. The blue box highlights the best case with optimum parameters selected from the experimental design.

For the network parameter optimization, we have taken a step-by-step approach described above. We first test a set of network parameters on AlexNet and SqueezeNet. Two sets of parameter optimization experiments are conducted. In the first test, the impact of the initial learning rate is tested on AlexNet and SqueezeNet using the half and the full data set of images. The experimental design approach runs several trial runs and tests the impact of each run on prediction accuracy, see Figure 10. The figure shows that validation accuracy is lower for smaller data sets and using a smaller value of initial learning rate gives better results for the full data set for both AlexNet and SqueezeNet.

**Figure 11:**
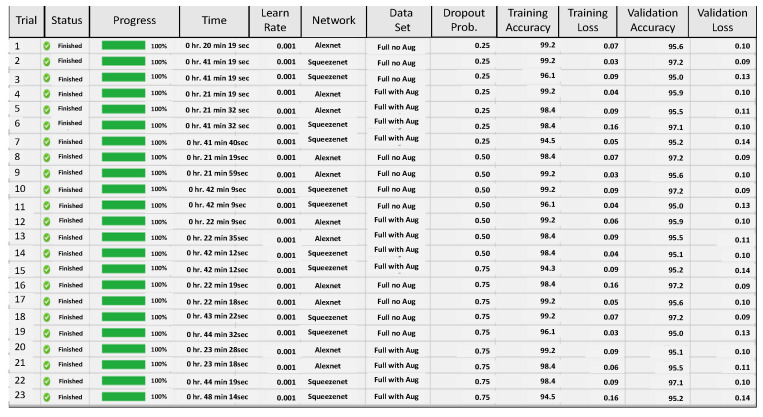
Figure showing application of network optimization workflow on AlexNet and SqueezeNet using the database of images and trial runs with Initial Learning rate and Dropout Probability variations.

In the next set of experiments, given the poor performance of the smaller data set, a full data set is used and the impact of two parameters, initial learning rate and network dropout probability, are tested simultaneously, on the validation accuracy of AlexNet and SqueezeNet. Again the experimental design approach runs several trial combinations and helps to suggest the best initial learning rate and the best network dropout probability, which will give the best accuracy, see Figure 11. Based on the output of these tests the network parameters are fine-tuned and then both networks are used for further analysis.

## 6. Color Recurrence Plot Analysis Using Transfer Learning

**Figure 12:**
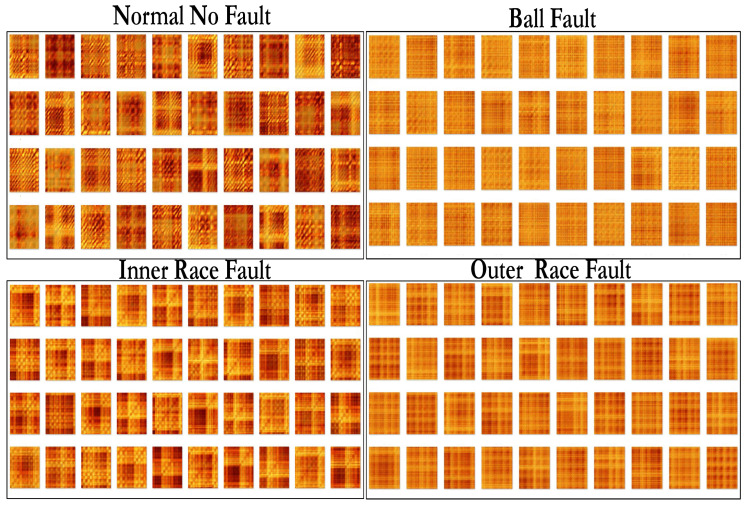
Images showing a subset of the images used for training the deep learning network.

For machine learning, one of the important steps is the preparation of data for training, testing and validation. For this purpose, the bearing fault data set described earlier in Section 4 is used. Averaged recurrence plots are created for normal data without bearing fault, drive end (inner race) bearing fault data and far end (outer race) bearing fault data set, see Figure 12, which shows some sample subset of images from each of the four data classes.

Initially, we used non-overlapping windows for creating averaged recurrence plots, which resulted in about 221 images for each category, but we realized that during the training and test that this data set was not adequate. Then to increase the number of averaged recurrence images we had to use overlapping windows on the data set with a window size of 550 data points, which results in 442 averaged images for each of the categories. The images are also converted into corresponding pixel resolution, 227 × 227, which would be suitable for AlexNet and SqueezeNet architecture.

Data is divided into two bigger sets comprising fan end and drive end data, where each set is further subdivided into four classes comprising normal, inner race, outer race and ball data sets. For classification, two problems are solved, first, where the subsets are kept intact and tested with normal data without any fault for the fan end and drive end individually, which results in a classification problem with four classes. The second classification task involved combining sub-classes of drive end and fan end, which results in 10 classes each for the fan end and drive end data set and therefore, a more challenging classification task.

**Figure 13:**
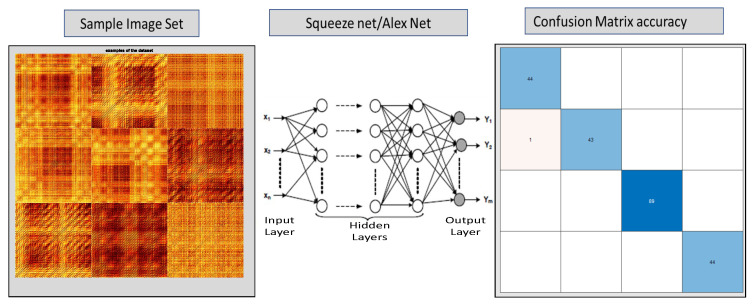
Figure showing conceptual workflow of how Alextnet or Squeezenet will be used for image classification using Color recurrence plots.

As described earlier in Section 5, once the data set was ready the transfer learning approach was used for carrying out recurrence plot analysis on the averaged image for each data set. A typical conceptual workflow of how the analysis could be carried out is shown in Figure 13. The figure shows how an image class was used to train the neural network such as Alexnet or Squeezenet using transfer learning and resulting confusion matrices are generated.

**Figure 14:**
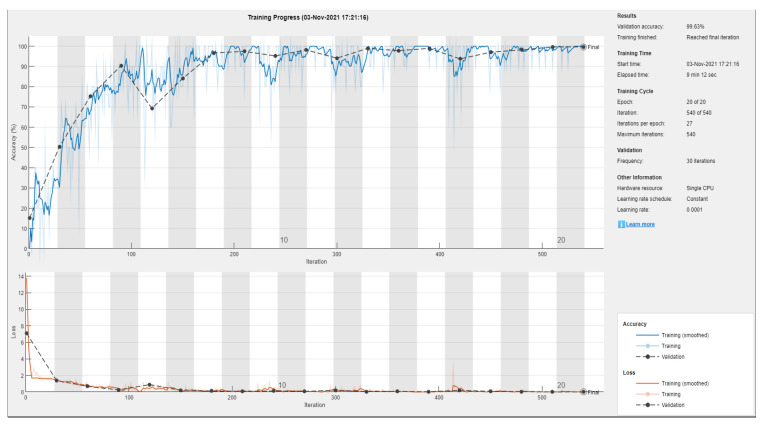
Figure showing training of SqueezeNet using Transfer learning on the Color recurrence plots combining the classes into a single classification problem.

**Figure 15:**
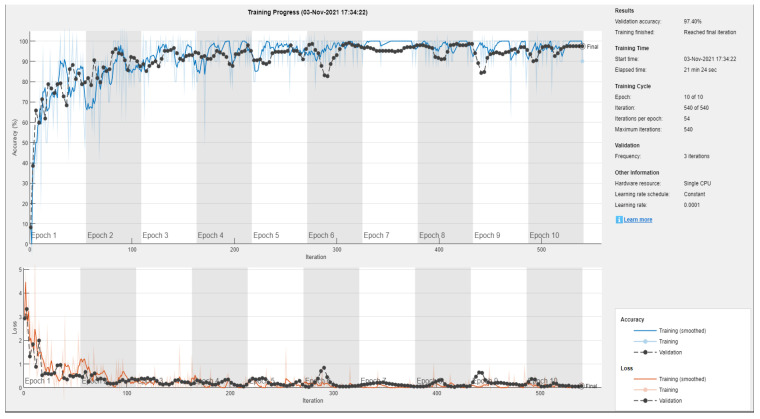
Figure showing training of AlexNet using Transfer learning on the Color recurrence plots combining the classes into a single classification problem.

For the first classification task, four subsets of the total data set were created for the drive end and fan end data set corresponding to data series any fault “normal”, inner race fault, outer race fault and ball bearing fault. Each subset comprised 442 images, which were divided into training, testing and validation using 80/20 ratios. Figure 14 and Figure 15 show the training metrics of the AlexNet and SqueezeNet, respectively. Once the network is trained it is then ready to be used for the classification task.

**Figure 16:**
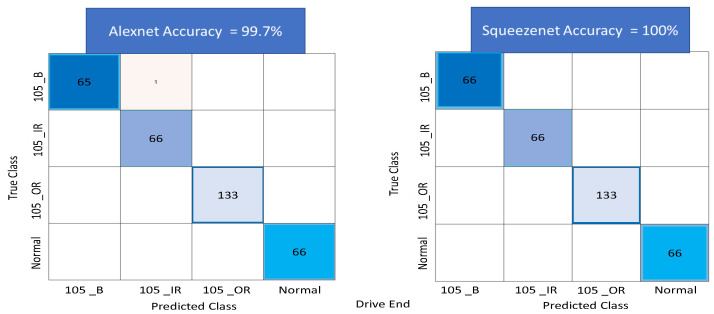
Figure showing Confusion Matrix for AlexNet and SqueezeNet using Transfer learning on the Color recurrence plots for the drive end bearing subset of the data set.

**Figure 17:**
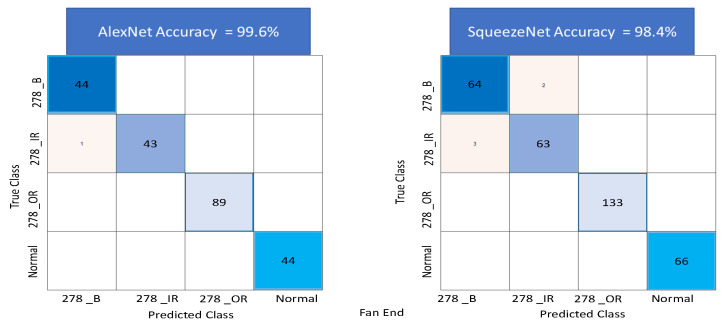
Figure showing Confusion Matrix for AlexNet and SqueezeNet using Transfer learning on the Color recurrence plots for the data set corresponding to the fan end bearing subset of the data set.

Several sensitivities were carried out to achieve high accuracy by adjusting some of the network parameters and the number of images used for training and testing. Particularly, the use of overlapping time series windows helped, to improve the initially lower accuracy rates, in achieving high accuracy rates with an increase in the number of images. The output of the classification algorithm in the form of a confusion matrix is shown in Figure 16, for the drive end data set. The figure shows the results of the test carried out using both AlexNet and SqueezeNet networks. A total of 99.7% accuracy is obtained for AlexNet and 100% accuracy is achieved for SqueezeNet. Figure 17 shows the confusion matrix using AlexNet and SqueezeNet for the fan end data set. A total of 99.6% accuracy is obtained for AlexNet and 98.4% accuracy is obtained for Squeezenet.

**Figure 18:**
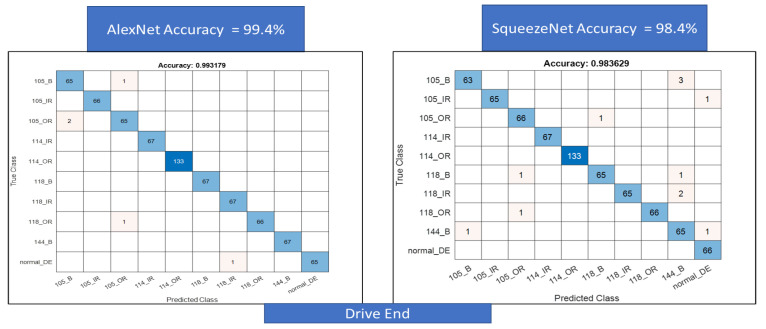
Figure showing Confusion Matrix for AlexNet and SqueezeNet using Transfer learning on the Color recurrence plots combining the classes into a single classification problem.

**Figure 19:**
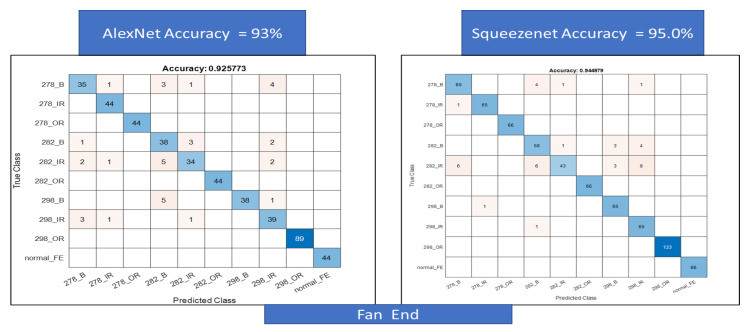
Figure showing confusion matrix using AlexNet and SqueezeNet for the fan end bearing data.

For the second classification task, we use a larger data set for a combined classification problem with 10 classes each for the fan end and the drive end data sets. First, the results for the confusion matrix generated using AlexNet and SqueezeNet for the drive end data set are shown in Figure 18. A total of 99.4% accuracy is achieved for AlexNet and 98.4% accuracy is achieved for SqueezeNet. Next, the same is performed for the fan end data set, accuracy, in this case, is lower at about 93% for AlexNet and 95% for SqueezeNet, respectively, see Figure 19.

## 7. Conclusions and Discussions

In this paper, a detailed analysis of bearing fault diagnosis is presented with the help of Color recurrence plots analysis. Standard recurrence plots are based on uniform time lags and are black and white. Whereas, the algorithms proposed in this paper use non-uniform time lags combined with an image averaging method, which results in unique Color recurrence plots. The Color recurrence plots make the analysis more interesting as the difference between different bearing types is enhanced through the use of color plots.

The recurrence plots are then analyzed with the help of an image classification approach using transfer learning, for which AlexNet and SqueezeNet are used. A large image database is created, from the data set using overlapping time series windows comprising 550 data points resulting in around 442 images for each class, which is used for training and testing of the networks using transfer learning. Network parameters are optimized based on an experimental design approach. The comparative results obtained using the image classification approach show a very high accuracy of around 99% accuracy for the two networks. The approach presented in this paper clearly demonstrates that recurrence analysis combined with machine learning forms a very powerful tool for early detection of bearing fault analysis using vibrations data.

Moreover, in this paper, only a simple averaging method is used to generate Color recurrence plots. It is possible to further explore other averaging methods such as harmonic or power law averaging and perform a comparative analysis of image classification between the different averaging methods. One of the aspects of averaging is that information is reduced due to averaging it could also be explored if only some specific projections could be averaged, which would lead to the creation of multiple groups of Color recurrence plots. It is an idea that needs further exploration and testing.

Additionally, in this paper, we have tested two extremes of the image classification task one with four classes, taking a subset of the data, and the other with 10 classes, taking a larger data set. It is possible to explore other intermediate groups of classes and further check the accuracy of the method proposed in this paper.

Finally, the novelty of the bearing fault diagnosis approach presented in this paper comes from two aspects. First, the use of Color recurrence plots, which, first have never been presented before, and second enhance the image patterns and help to significantly improve the classification task even in the case of bigger data sets where 10 classes are used for fan end and drive end data sets. The second aspect of novelty comes from the use of transfer learning, which does not require the design of a network from scratch but takes advantage of the existing networks, which have already been trained on a large data set of images. Although the second aspect is not completely new, its use in bearing fault diagnosis is new [17].

## Data Availability

Publicly available datasets were analyzed in this study. This data can be found here: [33].
